# Evidence for a Role of a Cortico-Subcortical Network for Automatic and Unconscious Motor Inhibition of Manual Responses

**DOI:** 10.1371/journal.pone.0048007

**Published:** 2012-10-24

**Authors:** Kevin D’Ostilio, Fabienne Collette, Christophe Phillips, Gaëtan Garraux

**Affiliations:** 1 MoVeRe group, Cyclotron Research Center, University of Liege, Liège, Belgium; 2 Department of Psychology, Cognition and Behavior, University of Liège, Liège, Belgium; 3 Cyclotron Research Center, University of Liège, Liège, Belgium; 4 Department of Electrical Engineering and Computer Science, University of Liège, Liège, Belgium; 5 Department of Neurology, University Hospital Center, Liège, Belgium; University of Montreal, Canada

## Abstract

It is now clear that non-consciously perceived stimuli can bias our decisions. Although previous researches highlighted the importance of automatic and unconscious processes involved in voluntary action, the neural correlates of such processes remain unclear. Basal ganglia dysfunctions have long been associated with impairment in automatic motor control. In addition, a key role of the medial frontal cortex has been suggested by administrating a subliminal masked prime task to a patient with a small lesion restricted to the supplementary motor area (SMA). In this task, invisible masked arrows stimuli were followed by visible arrow targets for a left or right hand response at different interstimuli intervals (ISI), producing a traditional facilitation effect for compatible trials at short ISI and a reversal inhibitory effect at longer ISI. Here, by using fast event-related fMRI and a weighted parametric analysis, we showed BOLD related activity changes in a cortico-subcortical network, especially in the SMA and the striatum, directly linked to the individual behavioral pattern. This new imaging result corroborates previous works on subliminal priming using lesional approaches. This finding implies that one of the roles of these regions was to suppress a partially activated movement below the threshold of awareness**.**

## Introduction

Response inhibition is one of the most widely investigated cognitive functions and is involved in a variety of tasks and processes [1]–[3]. This ability of suppressing a pre-potent response [4], mandatory to deal with a constantly changing environment, was traditionally associated with conscious control [5]. However, there is also evidence supporting the existence of another form of response inhibition that is more automatic and follows an unconscious motor activation phase. This type of inhibitory process has been mainly demonstrated using a specific masked prime task [6]–[8]. In this visuomotor task, participants are asked to make speeded button presses with the left or right hand following leftward or rightward pointing arrows, which are preceded by a subliminal masked prime arrow. When the interval between the mask and the target stimuli is short (ISI<80 ms), performance (i.e., reaction time (RT)) is better when prime and arrow stimuli point to the same direction (compatible trials) than in the opposite direction (incompatible trials). This has been traditionally labeled as a positive compatibility effect (PCE). Conversely, when this interval is longer (typically, 80 ms < ISI <200 ms), one may observe a Negative Compatibility Effect (NCE), namely a performance cost for compatible trials (longer RT, more errors) and a performance benefit for incompatible trials (shorter RT, fewer errors). This unexpected effect has been reproduced by many [9]–[13]. The NCE is often interpreted as resulting from an automatic (i.e. uncontrolled) and unconscious (i.e. triggered by subliminal stimuli) motor inhibition mechanism that suppresses the motor plan that was automatically and unconsciously activated by the prime arrow. According to this model, a conflict effect is elicited in the compatible condition when the previously suppressed motor plan has to be reactivated by the presentation of the target arrow.

While there is converging evidence for a role of the medial premotor cortex in the automatic activation of motor responses [14], [15], the neural correlates of the automatic and unconscious inhibition mechanisms remain poorly understood. The observations that such low-level process is early developed in children [16], but impaired in Huntington’s disease patients [11] and also in Parkinson’s disease patients [17], suggests that it is mediated by subcortical structures. In contrast, a role for the frontal cortex is supported by the observation that it has not been possible to demonstrate a NCE in patients with a focal lesion in medial premotor cortex [18]. Additionally, the automatic and unconscious inhibition seems to be dependant to the gamma-aminobutyric acid (GABA) concentration in this region [19].

The goal of the present study is to look for brain regions that are involved in the unconscious and automatic motor inhibition of hand movements using rapid event-related functional magnetic resonance imaging (fMRI) in combination with the subliminal masked prime paradigm. One important methodological issue inherent to this task is to dissociate the inhibition process from a conflict effect (see above). For instance, Boy et al. [20] used fMRI to look at the differences in activity between compatible and incompatible trials at ISI 150. They reported an increased brain activity in the supplementary motor area (SMA) for compatible trials. However, this differential activity is likely to be confounded by a conflict between response alternatives subsequent to the prime automatic inhibition that is present in both conditions. In a previous fMRI experiment, Aron et al. [11] were able to convincingly demonstrate an involvement of the caudate and the thalamus in low-level inhibition by comparing short (ISI 0) and long (ISI 150) intervals. However, the analysis was restricted to the basal ganglia leaving unexplored the relative contribution of other brain areas including the medial frontal cortex.

Here, we used a parametric analysis of BOLD signal changes at five different inter-stimulus intervals (ISI: 0, 100, 150, 200, 250 ms) weighted by individual RT performance. We believe that this methodological approach allowed isolating the automatic and unconscious inhibition process from the conflict effect while taking into account the inter-individual variability of compatibility effects [19]. At a behavioral level, we expected a positive compatibility effect (PCE) for the ISI 0, a reverse effect (NCE) at longer ISI (100, 150 and 200) and a positive rebound at much longer ISI (250) [21]. At a neuronal level, we predicted that our parametric statistical model would uncover brain areas where the temporal profile of BOLD signal changes matched that of behavioral data. Based on the results of previous studies (see above), we predicted that these brain areas would encompass the SMA and basal ganglia.

## Materials and Methods

### Ethics Statement

All procedures were executed in compliance with relevant laws and institutional guidelines. The study was approved by the Ethics Committee of the Faculty of Medicine, University of Liège, Belgium.

### Participants

A total of twenty-six right-handed healthy volunteers gave oral and written consent to participate in the study (11 men, mean age 22±2 years). None of the participants had any history of psychological or neurological disorders.

### fMRI Task Procedure

The behavioral paradigm consisted in a visuomotor RT task, as previously reported [22]. In brief, each trial of the masked prime task started with a fixation cross displayed on the center of the screen. Its display was pseudo-randomly jittered across trials between 1500 and 3000 milliseconds. After a blank screen of 300 ms, a prime stimulus, consisting in double leftward (<<) or rightward (>>) pointing arrows, was centrally presented for 17 ms at fixation. The prime was immediately followed by a mask stimulus displayed for 100 ms that consisted in 30 randomly oriented lines covering a rectangular area centered on the prime display area. Following this backward mask stimulus, a target stimulus, whose physical properties were identical to that of the prime stimulus, was presented for 100 ms in the centre of the screen. The direction of the target stimulus was either identical (compatible condition) or opposite (incompatible condition) to the prime stimulus. Participants were asked to respond to the target stimuli as quickly and as accurately as possible by pressing a button with the corresponding hand. All stimuli subtended a visual angle of approximately 1.5°×1° and were displayed at the center of the screen. Visual stimuli were generated and subject responses recorded by a personal computer using COGENT Cognitive interface software (COGENT 2000, Wellcome Department of Imaging Neuroscience, London, UK) implemented in Matlab 6.1 (Mathworks, Sherborn, MA). Here, there were five main experimental conditions defined by the time elapsed between the mask display onset and the target display onset: 0, 100, 150, 200 and 250 ms. The 0 ms interstimuli interval (ISI) condition was identical to that previously reported [22]. Longer ISIs were obtained by modulating the time duration of a blank screen presented between the mask display offset and the target display onset.

**Figure 1:**
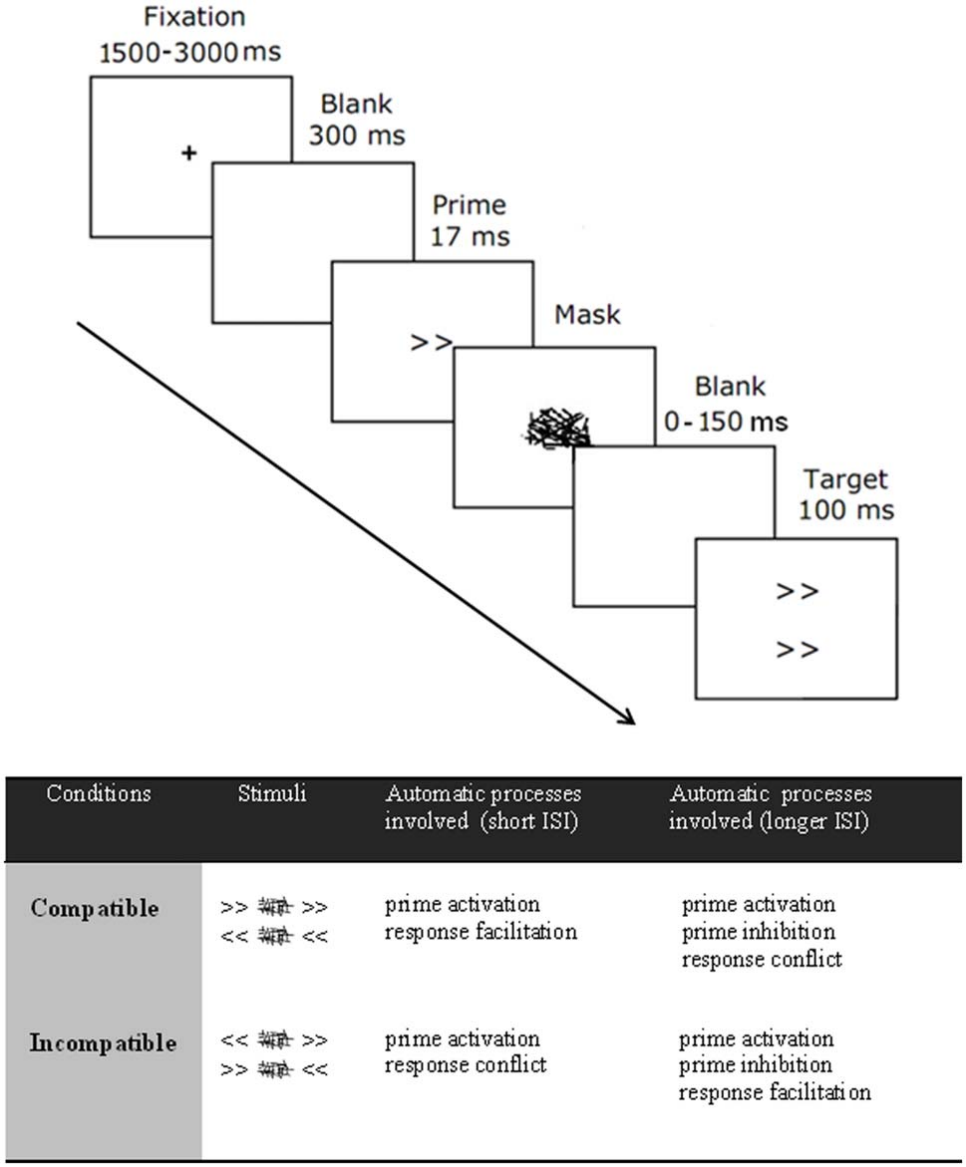
Task paradigm. Top: Behavioral task paradigm for a compatible trial. The duration of the blank screen before the arrow target (0–50–100–150 ms) determined the ISI (100–150–200–250). In the ISI-0, targets appeared together with the mask. Bottom: summary of the processes induced by the task at short (i.e. 0 ms) and longer ISIs (i.e.150 ms).

The experiment consisted in two fMRI sessions (ISI-0 and ISI-100–150–200–250), presented in a randomized order across subjects. Prior to the fMRI experiment, each subject was trained on a practice block of 60 trials outside the MR scanner. In the scanner, each subject performed 144 randomized trials for the ISI-0 and 240 trials for the long ISI (100–150–200–250), including 24 no-response trials (masked prime but no target stimuli) and 24 null events (fixation cross display only without any arrow or mask stimuli). After completion of a block of 36 trials, subjects were provided with a 20 sec. rest period during which the mean global RT during the last performed block was displayed. Figure 1 illustrates the behavioral task paradigm, along with a summary of the induced automatic processes underlying each condition.

### Prime Identification

After the main fMRI experiment, participants were administered a prime identification task in the MRI scanner to assess the level of prime perception as a function of the prime display duration. The experimental set up was the same as during the main experiment including the fact that the scanner was running. In each trial, the stimuli (left or right pointing arrow) and their temporal sequence of presentation were the same as in the main experiment with the exception that the target stimulus was a question mark displayed from 1 to 1.7 seconds after the mask. This question mark prompted the participants to make a response with the left or right hand within 3 seconds. Prime display duration varied according to a one-down/two-up staircase procedure [16]. The task always started with a 167 ms display trial. In the following trials, prime display was shortened by steps of 17 milliseconds whenever participants gave a correct response and prolonged by 34 milliseconds after an incorrect response. In trials where the prime was not consciously perceived, participants were invited to guess the response they felt the most accurate.

### Imaging Data Acquisition

BOLD fMRI data were acquired on a 3 Tesla scanner (Siemens, Allegra, Erlangen, Germany) using a T2* sensitive gradient echo EPI sequence (TR = 1170 ms, TE = 30 ms, FA = 90°, matrix size 64×64×20, voxel size = 3.4×3.4×5 mm3). These parameters allow recovering 91% of the BOLD signal despite the relatively short TR. Twenty 5-mm-thick slices were acquired, covering nearly the whole brain. For each session, the first eight volumes, acquired before stimulus presentation, were discarded to allow for T1 saturation effects. Head movement was minimized by restraining the subject’s head using a vacuum cushion. Stimuli were displayed on a screen positioned at the rear of the scanner, which the subject could comfortably see through a mirror mounted on the standard head coil. A high resolution structural images was obtained in all participants using a T1-weighted 3D MDEFT sequence (TR = 7.92 ms, TE = 2.4 ms, FA = 15°, matrix size = 224× 256 × 176, voxel size = 1×1×1 mm3).

### Behavioral Data Analysis

Mean (RT) were calculated for each ISI condition. Trials with incorrect responses and RT longer than 1 second were discarded from the RT analysis. A repeated measures ANOVA was performed on mean RTs for the factor prime-target ISI (0, 100, 150, 200, 250) and compatibility (compatible, incompatible) with STATISTICA 9.1, statsoft, France. As we had strong *a priori* hypothesis, planned comparisons (i.e. not corrected for multiple comparisons) were used to compare compatible and incompatible RTs at each ISI.

In the identification task, we calculated the mean prime duration by averaging all prime durations for each participant after having discarded the first 10 trials, as this is the minimum number of steps needed to reach the 17-ms performance limit [16] in order to obtain a converging value of the display duration at which subjects consciously perceive the masked prime on average. We also assessed the mean response accuracy of 17 ms trials and tested if this was significantly different from performance at chance level using a t-test [11].

### Imaging Data Analysis

Data were preprocessed and analyzed using SPM8 (Wellcome Trust Centre for Neuroimaging, http://www.fil.ion.ucl.ac.uk/spm) implemented in MATLAB 7.4.0 (Mathworks Inc., Sherbom, MA). For each participant, we first applied a slice-timing correction to compensate for the staggered order of slices acquired by EPI. The BOLD time series was then spatially realigned using rigid body transformations that minimize the residual sum of square between the first and each subsequent image. The mean EPI image created from the realigned time-series was spatially coregistered to the anatomical MRI image and coregistration parameters were applied to the realigned BOLD time series. The individual anatomical MRI was spatially normalized into the MNI space (Montreal Neurological Institute, http://www.bic.mni.mcgill.ca) using the unified segmentation approach [23], and normalization parameters were subsequently applied to the coregistered BOLD times series, then resliced to a voxel size of 2×2×2 mm^3^, and spatially smoothed using an 6 mm FWHM Gaussian kernel.

In first-level SPM analyses, all main experimental trials (compatible, incompatible, no-response and null events) were separately modeled as single events time-locked on the target stimulus display onset. Each event was convolved with a canonical hemodynamic response function (HRF) and its time and dispersion derivatives. In addition, the statistical model included 6 additional regressors representing the realignment parameters from the rigid body-transformation step. A high pass filter using a cut-off period of 128 seconds was applied in order to remove the low frequency drifts from the time series. Serial autocorrelation was accounted for by a first-degree autoregressive model plus additive white noise. The session effect was modeled in the design matrix by means of two separate regressors. Parameter estimates and variance were derived voxel-by-voxel and the main effect of each condition was assessed using t-contrasts.

We constructed first-level individual contrasts in order to isolate the automatic and unconscious inhibition effect whatever the prime-target relationship. To this end, we used a parametric analysis weighted by the profile of behavioral responses across ISIs. The contrast modeling the effect of each ISI in the design matrix was weighted according to the size of the corresponding compatibility effect. The simplest approach is to apply a mean-corrected weighting procedure. Under the null hypothesis of no difference, the weightings of ISI regressors will add up to zero. One form of this equation is as follow: CE1+×+ CE2+×+ CE3+×+ CE4+×+ CE5+× = 0. For instance, the size of the compatibility effects in subject number #3 showed the following profile: 27 ms, −57 ms; −30 ms; −17 ms, and 5 ms at ISI 0, 100, 150, 200 and 250, respectively. Here, × = 14.4, and the corresponding regressors were weighted by a value of 41.4; −42.6; −15.6; −2.6; 19.4, respectively. The output of these individual first-level analyses was a contrast image representing the individual BOLD response with respect to each ISI.

Contrasts images from all participants were entered into a second-level random effect one-sample t-test analysis. We reported activations in regions defined by our a priori hypotheses: basal ganglia and frontal cortex. Significance level of resulting SPM maps was first set at a p<0.001 uncorrected. We additionally performed multiple comparison corrections using permutation testing as implemented in the threshold free cluster enhancement toolbox (TFCE r56 available at http://dbm.neuro.uni-jena.de/tfce). We performed 5000 permutations and set the significance threshold to p = 0.05 corrected for multiple comparisons at the cluster level using the family wise error (FWE) procedure [24], considering as a very safe approach in order to minimize the effect of non-stationarity in the inference [25].

## Results and Discussion

### Behavioral Results

We have evidence suggesting that the 17 ms prime stimuli used in the main fMRI experiment were not consciously perceived. In the one-down/two-up staircase prime identification task [16] administered after the fMRI experiment, the group mean prime duration was 36 ms, suggesting this was the threshold at which participants can just consciously perceive the prime. Additionally, when looking at 17-ms prime duration trials, the group mean response accuracy was 46%. A t-test showed that this result was not significantly different from chance levels (p>0.05).

**Figure 2:**
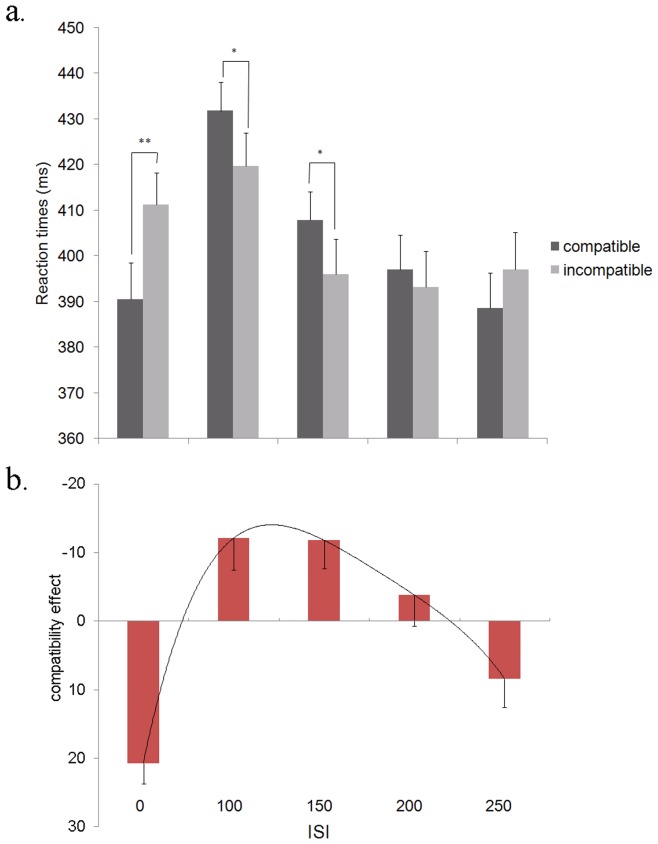
Behavioral results. **a.** Group mean RTs in compatible and incompatible trials showed the expected pattern with a PCE at ISI-0 followed by a NCE at ISI-100 and ISI-150. At ISI-200, the size of the NCE decreased and did not reach the level of significance anymore. At ISI-250, there was a tendency toward a PCE. **b.** Group mean compatibility effects (i.e. mean RT in incompatible trials – mean RT in compatible trials) across all ISIs. Vertical bars represent standard errors.

In the main fMRI experiment, we replicated the masked priming effects [6]. The repeated measures ANOVA of behavioral data (global interaction ISI*compatibility: F(4, 100) = 16.54, p<0.001), showed faster RT (i.e., motor response facilitation) in compatible than incompatible trials at 0-ISI (PCE: diff = 21 ms, *t*(25) =  −7, p<0.001) and the reverse (NCE) at 100 (diff = −12 ms, *t*(25) = 2.62, p = 0.015) and 150-ISI (diff = −12 ms, *t*(25) = 2.88, p = 0.008). At 200 and 250-ISI, we no longer found significant compatibility effects (p>0.05) (Figure 2). Accuracy rate showed a similar tendency as RT but no statistical inference was made given the small number of errors (data not shown).

Several models have been proposed to account for the NCE, which is specifically observed when the target stimulus is displayed 100 or 150 ms after the mask onset display (Figure 1). Some authors argued for perceptual processes such as interactions between geometric features in prime and mask [26], [27] or repetition blindness [28]. However, the fact that more recent studies used masks composed of random lines or demonstrated a NCE even when no target stimuli were presented after the mask [29] suggested another explanation of the NCE: a rapid motor inhibition, which could be either self-triggered [10], [30] or masked-triggered [31], [32]. The results of the fMRI data analysis presented below provide novel evidence suggesting that the NCE is mediated at the motor preparation stage involving the basal-ganglia motor loop, downstream perceptual processes.

### fMRI Results

fMRI data were analyzed to model brain activity changes that closely match compatibility effects across ISIs. BOLD fMRI activity was matched to the RT pattern on a subject-by-subject basis by weighting the ISI regressors in the design matrix according to the size of the compatibility effect at each ISI. This allows us to model the NCE over and above any conflict effect embedded in the task at 100 and 150 ms (see introduction section). Three subjects were discarded from this analysis because they did not show the expected behavioral pattern of a PCE followed by a NCE as a function of increased ISI.

**Figure 3:**
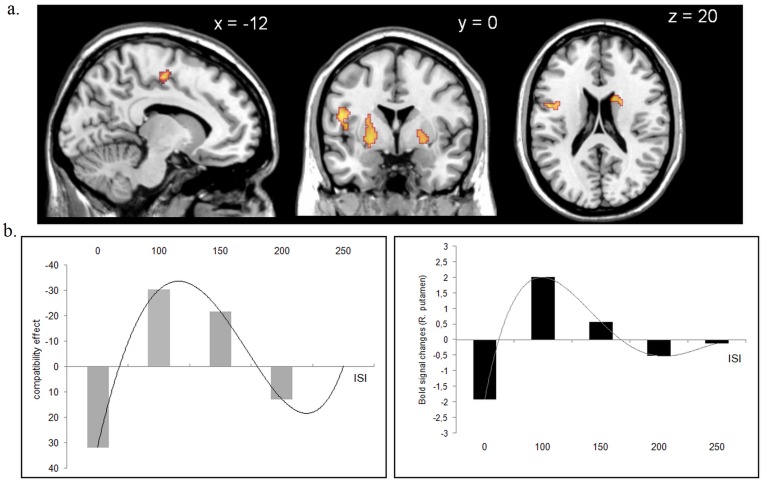
Results of the weighted parametric fMRI analysis. **a.** The behavioral pattern of PCE/NCE was associated with a similar pattern of activity changes in the premotor cortex, especially the SMA, the caudate and the putamen (p<0.005 for display purpose). **b.** Comparison between the compatibility effect and striatal BOLD signal activity across ISIs in a representative subject.

Results provide novel evidence for a role of the basal-ganglia and premotor cortices in unconscious and automatic inhibition of voluntary actions. Although the contrast assessing stronger activations for ISIs with PCE (i.e. ISIs with PCE > ISIs with NCE) did not reveal any significant voxels at this threshold, the opposite comparison (i.e. ISIs with NCE > ISIs with PCE) showed greater activity in the bilateral striatum, the left precentral gyrus and the posterior part of the left SMA (Table 1 and figure 3). At a lower threshold (i.e. p = 0.005 uncorrected), a stronger activation of the right SMA was also observed (MNI coordinates: 10, −14, 52).

**Table 1:** Brain regions showing a significant increase in BOLD signal following the behavioral pattern of facilitation/inhibition.

Anatomical regions	Voxel Z value	Voxel p(unc)	Coordinates x,y,z (mm)
Right putamen*	4.00	0.00003	24 −10 12
Left precentral gyrus*	3.58	0.0002	−48 2 12
Right caudate*	3.23	0.0006	16 8 22
Left supplementary motor area*	3.21	0.0007	−12 −14 52
Left pallidum/putamen	3.11	0.0009	−24 0 −2

* p≤0.05 corrected for multiple comparisons using TFCE

Our experimental design allows demonstrating for the first time that the NCE was specifically related to activity changes in cortico-subcortical motor circuits. The neural correlate of the NCE has been tentatively addressed in two previous fMRI experiments during the masked prime paradigm. The experimental design used by Aron et al. [11] allows dissociating motor response inhibition from the conflict effect at ISI-150. In that study, fMRI data analysis was restricted the basal ganglia and thalamus. The main finding was a strong relationship between BOLD activity changes in the striatum and motor response inhibition at ISI-150, over and above a simple conflict effect. However, results showed that motor response inhibition was related to a relative decrease in activity in these regions rather than an increase as in the present study. This apparent discrepancy may be explained by fundamental differences in the statistical model used for fMRI data analysis. Indeed, in [11], results were obtained from an interaction analysis comparing ISI-0 with ISI-150 conditions by subtracting each condition with neutral trials (with target but no directional prime) beforehand in order to suppress the ISI perceptual-effect. In another fMRI study, Boy et al. [20] directly compared BOLD activity in the medial premotor cortex between compatible and incompatible trials at ISI-150. The authors found a difference in the SMA but not in the pre-SMA. However, their behavioral paradigm did not allow them separating conflict effects from motor response inhibition at ISI-150 (see table at the bottom of figure 1).

In the model of basal ganglia-thalamo-cortical loops, the striatum and premotor cortices are considered as key structures of the motor loop. In this model, the putamen receives excitatory glutamatergic inputs from the premotor cortex, including the SMA [33], [34]. The exact function of the basal ganglia still need to be elucidated but they are known to play an important role in the control of movement execution, notably in initiating, inhibiting, switching behaviors, and also in the processing of rewards and other feedback [34]. Mink [35] proposed that basal ganglia are crucial in controlling the balance between movements facilitation and suppression. Furthermore, activations of the striatum obtained in the present fMRI experiment are in agreement with previous studies using lesional approaches. Indeed, deficits in unconscious and automatic inhibition are supported by behavioral observations in patients with basal ganglia disorders [11], [17], [36] and also in patients with lesion of the SMA [18]. Altogether, it is possible that the mechanisms by which the cortico-striatal circuits contribute to motor control are automatic and unconscious activation/inhibition of motor plans through an efficient sensorimotor integration [37], suggesting that some disorders of motor control might result from a disturbance of this balance.

Results from this and previous studies suggest that the medial premotor cortex, including the SMA is involved in both the PCE and NCE. Indeed, using the prime masked task, we previously demonstrated increased BOLD activity in the medial premotor cortex in response to visual stimuli presented below the threshold of awareness and in the absence of any motor response. This effect was observed only for visual stimuli that had been previously associated with a specific motor response (i.e. arrow stimulus) and not for neutral stimuli (i.e. that was not associated with a specific motor response such as a fixation cross stimulus) [15]. It has been suggested that this kind of automatic sensorimotor processes forms an intrinsic part of all behavior, rather than being totally separated from voluntary action and being paradoxically greater when many sensorimotor associations are in competition because every stimulus will trigger a representation of the associated motor response [38]. Recently, this hypothesis has received some supports from the results of Boy et al. [39] who combined the masked prime task with a traditional flanker task. The results showed that flanker interference interacted with the automatic and unconscious inhibition suggesting an overlap between conscious cognitive control and subliminal negative priming. In a subsequent study, we found that the unconscious motor response facilitation at ISI-0 was also associated with activity changes in the SMA, especially with a deactivation of this region as a result of a repetition suppression of the fMRI BOLD signal in compatible trials [22]. Thus, the SMA seems to play an important role in several unconscious processes elicited by the subliminal presentation of visual stimuli that have been previously associated with a specific motor response. First, at the prime level, the SMA may be involved in the automatic stimulus induced activation of the corresponding motor plans. Second, if the ISI reaches a certain threshold, the activity in this region will increase, mediating the suppression of the motor plan activation elicited by the prime. At the target level, the SMA activity might be reduced as a result of a facilitation effect. Unfortunately, the temporal resolution of fMRI does not allow dissociating these distinct roles of the SMA.

A central role of SMA in automatic and unconscious motor response activation and inhibition is also supported by results from other experiments. This region has been previously associated with the Bereitschaftspotential or readiness potential, an early negativity preceding movement [40] and also conscious intention to movement [41]. As movements are first initiated unconsciously in the SMA [42], [43], the feeling of being in control should be a reconstructed subjective experience [44]. But according to Libet [45], our freedom may reside in the ability to stop a movement after it becomes conscious. However, other researchers demonstrated that a veto cannot be consciously initiated by combining a stop signal and an intentional action paradigm [44]. It is therefore possible that this “veto” power or “free won’t” is not more than an automatic inhibitory process that also notably takes place in the SMA, challenging the traditional view of the need of consciousness in voluntary control of action.

Prefrontal and premotor cortices may play different roles during inhibition. As the former has been involved in impulse inhibition that prevents premature initiation of a planned response, the latter has been preferentially associated with competitive processes that occur during selection helping to specify what response should be produced [46], [47]. We previously demonstrated that response conflict induced by subliminal stimuli is associated with stronger activity in the anterior cingulate cortex and the dorsolateral prefrontal cortex [22]. Here, the inhibition induced by the prime recruited a part of the premotor cortex but no cognitive area, suggesting that this unconscious and automatic inhibition is a motor process allowing preparatory mechanisms to automatically suppress an activated movement without the need of cognitive processes. Taking together, these results challenge the view of a conscious dependence to control action. Therefore, we proposed that the automatic and unconscious inhibition in the subliminal masked prime task is a “pure” inhibitory process, (i.e. without contamination because it cannot respond to rules in working memory). Traditional inhibition tasks do not solely require inhibitory processes because they are usually contaminated by task instructions held in working memory and so by other linked high-level cognitive processes [48].

The limitation of this study concerns the task design in itself. It is possible that a least questionable way of data interpretation would be to include neutral response trials. However, the use of a weighted analysis that compares BOLD signal between ISIs, whatever the compatibility of the prime-target relationship, as well as the statistical correction for multiple comparisons can reassure on the result validity of the present experiment.

In summary, this fMRI study shows for the first time activity changes in the SMA and the striatum that closely match the negative compatibility effect during visuo-motor priming task. This novel result provides support for current models of visuo-motor control that predict a role for these areas in the automatic motor response inhibition that follows the automatic motor response activation elicited by the presentation of a visual stimuli below the threshold of awareness.
